# LaTe_1.9_: a tenfold superstructure of the ZrSSi type

**DOI:** 10.1107/S2056989022004844

**Published:** 2022-05-13

**Authors:** Hagen Poddig, Thomas Doert

**Affiliations:** a Faculty of Chemistry and Food Chemistry, TU Dresden, D-01062 Dresden, Germany; Vienna University of Technology, Austria

**Keywords:** crystal structure, telluride, rare earth metal, superstructure, twinning

## Abstract

Single crystals of LaTe_1.9_ (lanthanum telluride) have been obtained by chemical transport reactions with iodine as transport agent. LaTe_1.9_ adopts the CeSe_1.9_ structure type and crystallizes in the space group *P*4_2_/*n* with lattice parameters *a* = 10.1072 (3) Å and *c* = 18.2874 (6) Å. The crystal structure comprises an alternating stacking of puckered [LaTe] slabs and planar [Te] layers along [001]. The planar [Te] layer is dominated by dumbbell-shaped Te_2_^2−^ anions along an isolated Te^2−^ anion and a vacancy. The Te_2_^2−^ anions form an eight-membered ring enclosing the vacancy in the centre.

## Abstract 2

The crystal structure of LaTe_1.9_ (CeSe_1.9_ structure type; space group *P*4_2_/*n*) was determined from a twinned crystal. LaTe_1.9_ features puckered [LaTe] slabs and planar [Te] layers consisting of Te_2_^2−^ and Te^2−^ anions.

## 1. Chemical context

Chalcogenides *REX*_2–*δ*_ (*RE* = Y, La–Nd, Sm, Gd–Lu; *X* = S, Se, Te) of trivalent rare-earth metals comprise a large structural variety in a small compositional range 0 ≤ *δ* ≤ 0.2. This variety can mainly be attributed to the amount of vacancies as this strongly affects the final structural motif (Doert & Müller, 2016 ▸). All crystal structures share a common motif of alternating [*REX*] and planar [*X*] layers, related to their common aristotype, the structure of ZrSSi. Here, the same stacking arrangement is observed with a puckered [ZrS] slab and a planar [Si] layer, where an idealized square-planar [Si] layer is realized (Onken *et al.*, 1964 ▸; Klein Haneveld & Jellinek, 1964 ▸). The chalcogenides, however, do not form a square-planar arrangement for electronic reasons, which can be understood by their charge-balanced formula: considering trivalent rare-earth metal cations only, the puckered [*REX*] slab bears a single positive charge per formula unit, which needs to be compensated by atoms of the planar [*X*] layer. This is achieved by forming dinuclear *X*_2_^2−^ anions in the stoichiometric dichacolgenides *REX*_2_. The formation of such dumbbell-shaped anions results in a distortion from the ideal square-planar layer. Reducing the chalcogenide content results in the formation of vacancies inside the planar [*X*] layer, which in turn forces a reaction of the remaining atoms to balance the missing charge. Consequently, an isolated *X* ^2−^ anion per vacancy is formed to maintain a charge-balanced motif, adding two new constituting fragments to the planar layer. As vacancies are not randomly distributed within the layer, commensurate and incommensurately modulated superstructures are found (Doert & Müller, 2016 ▸). The structural chemistry of the corresponding sulfides and selenides has been thoroughly investigated, revealing several crystal structures that are observed for both chalcogens. The tellurides, however, do not always match the structures of their sulfur and selenium congeners, as shown for LaTe_2_ (Stöwe, 2000*a* ▸), CeTe_2_ (Stöwe, 2000*b* ▸) and PrTe_2_ (Stöwe, 2000*c* ▸). Discrepancies are also observed for the Te-deficient compound NdTe_1.89 (1)_ (Stöwe, 2001 ▸). However, the CeSe_1.9_ type (Plambeck-Fischer *et al.*, 1989 ▸) with a 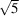 × 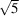 ×2 supercell of the basic ZrSSi structure seems common to sulfides, selenides and tellurides. CeTe_1.9_ was found to adopt this superstructure in space group *P*4_2_/*n* (No. 86) (Ijjaali & Ibers, 2006 ▸). The general motif of alternating stacks of [*RE*Te] slabs and planar [Te] layers is preserved in this structure, the planar [Te] layer comprise four Te_2_^2−^ anions surrounding a vacancy, resembling an eight-membered Te ring with alternating long and short distances. Four of these Te rings surround an isolated Te^2−^ anion in a pinwheel-like arrangement. Rationalizing this motif yields ten negative charges due to four Te_2_^2−^ and a single Te^2−^ anion, balancing ten positive charges of each [*RE*Te] layer. Here we report on the isotypic compound LaTe_1.9_, for which no structural characterization has been published yet.

## 2. Structural commentary

**Figure 1:**
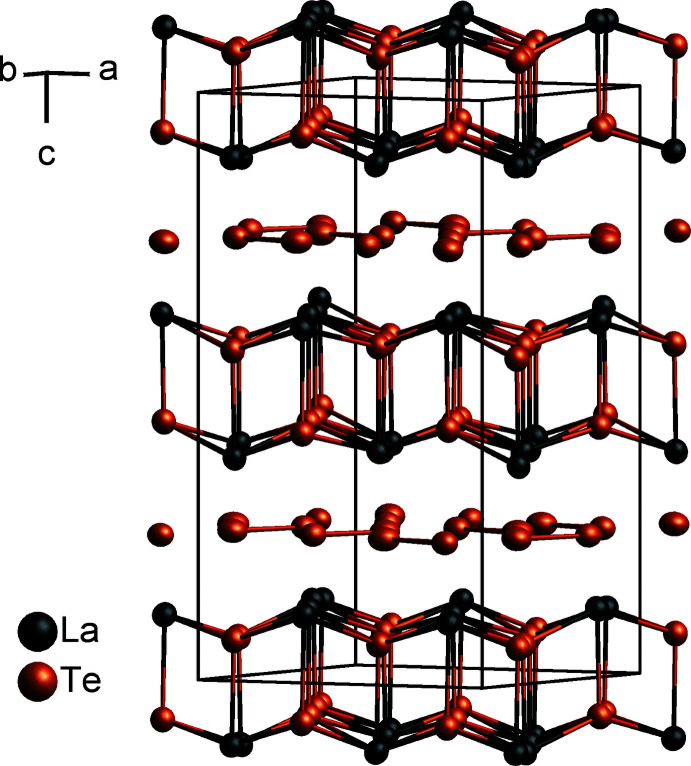
Crystal structure of LaTe_1.9_ with displacement ellipsoids drawn at the 99.95% probability level. The stacking arrangement of puckered [LaTe] slabs and planar [Te] layers along [001] is shown.

**Figure 2:**
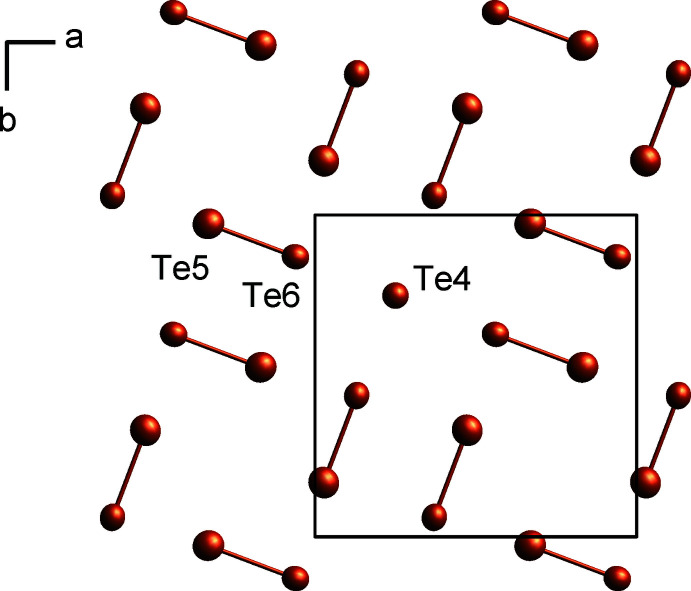
[Te] layer of LaTe_1.9_ with four Te_2_^2−^ anions enclosing a vacancy each and surrounding an isolated Te^2−^ anion; displacement ellipsoids are drawn at the 99.95% probability level.

LaTe_1.9_ crystallizes in space group *P*4_2_/*n* (No. 86) in the CeSe_1.9_ structure type (Plambeck-Fischer *et al.*, 1989 ▸) with *a* = 10.1072 (3) Å and *c* = 18.2874 (6) Å, corresponding to a 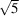 × 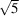 ×2 superstructure of the basic ZrSSi unit cell. As indicated above, two stacks of the basic arrangement are present in the structure of LaTe_1.9_ as the Te-deficient planar [Te] layers are shifted by an *n*-glide against each other (Fig. 1 ▸). The La atoms are coordinated by eight Te atoms (La2), respectively nine Te atoms (La1, La3) forming a bicapped, respectively a tricapped trigonal prism. The La—Te distances within the slabs range from 3.2637 (2) to 3.3594 (2) Å and from 3.2944 (3) to 3.4480 (3) between the planar [Te] layer and La. Calculating the bond-valence sum *bvs* (Brese & O’Keeffe, 1991 ▸) for each La site results in 2.99 valence units (v.u.) for La1, 3.06 v.u. for La2 and 2.94 v.u. for La3, which are all very close to the expected value of +3 considering the previously discussed charge-balancing situation. The tellurium layer exhibits a pinwheel-like arrangement of four eight-membered Te squares surrounding a single Te^2−^ anion in its centre (Fig. 2 ▸), common to all compounds of the CeSe_1.9_ type.

In view of the alternating short and long distances, the Te ring can be understood as being built up from four dinuclear Te_2_^2−^ anions enclosing a vacancy with alternating bonding and non-bonding distances of 2.9224 (3) and 3.1413 (3) Å, respectively.

In accordance with the charge balancing mentioned above and *Z* = 20, a structured formula of LaTe_1.9_ can be written as [(La^3+^)_20_(Te^2−^)_20_] [(Te_2_^2−^)_8_(Te^2−^)_2_]. This easily explains the anionic motifs and their qu­antity in the planar [Te] layer: Te5 and Te6 (both on *Wyckoff* site 8*g*) form the dumbbell-shaped Te_2_^2−^ anions whereas Te4 (*Wyckoff* site 2*b*) represents the isolated Te^2−^ (Fig. 2 ▸).

## 3. Database survey

The CeSe_1.9_ structure type (Plambeck-Fischer *et al.*, 1989 ▸) is realized by several rare-earth metal sulfides and selenides but only by a few tellurides, CeTe_1.9_ being one prominent example (Ijjaali & Ibers, 2006 ▸). The inter­atomic distances of LaTe_1.9_ match those observed for CeTe_1.9_ quite well, including the bonding and non-bonding distances in the planar [Te] layers [2.9224 (3) Å and 3.1413 (3) Å in LaTe_1.9_ *vs* 2.9194 (5) Å and 3.1204 (5) Å in CeTe_1.9_]. However, the bonding Te—Te distances in these two compounds are considerably longer compared to compounds featuring (largely) isolated Te_2_^2−^ anions as constituting fragments, *e.g.* in *α*-K_2_Te_2_ (2.86 Å), *β*-K_2_Te_2_ [2.790 (1) Å], Rb_2_Te_2_ (2.78 Å) or GdTe_1.8_ [2.868 (1) Å] (Böttcher *et al.*, 1993 ▸; Poddig *et al.*, 2018 ▸).

## 4. Synthesis and crystallization

Crystals of LaTe_1.9_ were found as a byproduct during the investigation of the system La–Te in chemical transport experiments using iodine as transport agent. All preparation steps were carried out in an argon-filled (5.0, Praxair Deutschland GmbH, Düsseldorf, Germany) glove box (MBraun, Garching, Germany). Starting from the elements, 300 mg of a stoichiometric mixture of La (99.5%, MaTecK) and Te (Merck, > 99.9%, reduced in H_2_ stream at 670 K) were ground and loaded into a silica ampule. A small amount of I_2_ (Roth, > 99.8%, purified by sublimating twice prior to use) was added inside the glove box before flame-sealing the ampule under dynamic vacuum (*p* ≤ 1×10^−3^ mbar). The ampule was heated with a ramp of 2 K min^−1^ to 1173 K before applying a gradient from 1173 → 1073 K, where the actual transport took place. After seven days, the ampule was cooled down to room temperature. A synthesis resulting in a phase pure product of LaTe_1.9_ has not yet been successful. The reason is most probably that two other Te-deficient compounds also exist in the composition range LaTe_2–*δ*_ (0 ≤ *δ* ≤ 0.2) along the stoichiometric ditelluride, namely LaTe_1.94 (1)_ and LaTe_1.82 (1)_ (Poddig & Doert, 2020 ▸; Poddig *et al.*, 2020 ▸). To address the stability ranges of the individual phases, the chalcogen vapor pressures and temperatures have to be evaluated and controlled precisely during synthesis (Müller *et al.*, 2010 ▸).

## 5. Refinement

**Figure 3:**
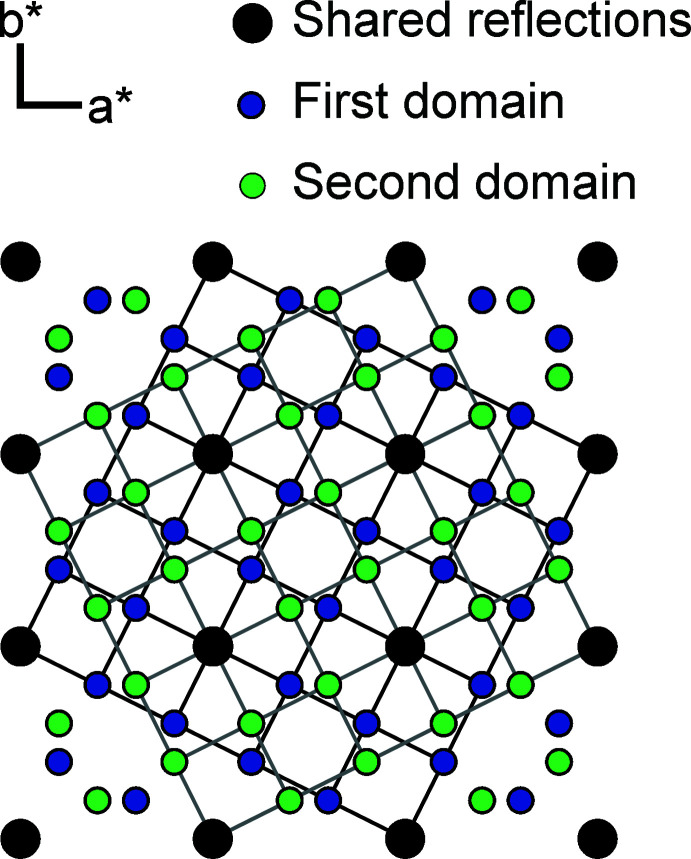
Projection of the X-ray diffraction pattern of a twinned crystal of LaTe_1.9_ along [001]. The individual reflections of the two domains are indicated as green and blue dots, coinciding reflections are marked in black. The axes correspond to the basic structure.

Crystal data, data collection and structure refinement details are summarized in Table 1 ▸. All investigated crystals of LaTe_1.9_ were found as reticular merohedric twins with a twin index *n* = 5. On a first glance, the diffraction patterns seem to suggest a large tetra­gonal unit cell with apparent lattice parameters of *a* = 22.6211 (6) Å and *c* = 18.3135 (5) Å, corresponding to a 50-fold superstructure of the basic ZrSSi structure (Fig. 3 ▸). Similar apparent supercells have been reported for the sulfides SmS_1.9_ (Tamazyan *et al.*, 2000 ▸) or TmS_1.9_ (Müller *et al.*, 2012 ▸), and can be explained by twinning along the mirror planes in (100) and (110) of the twin lattice. A schematic scheme drawn along [001] is depicted in Fig. 3 ▸, illustrating the lattices of each domain. The corresponding twin law calculated by the diffractometer software (Bruker, 2016 ▸) corresponds to the twin law derived for SmS_1.9_ (0.6 −0.8 0 −0.8 −0.6 0 0 0 −1). Both domains were handled during the process of integrating and correcting the data, and the refinements were performed on a HKLF5 format file. The twin ratio of the two domains calculated by *SHELXL* is 0.57 (1):43 (1).

## Supplementary Material

Crystal structure: contains datablock(s) global, I. DOI: 10.1107/S2056989022004844/wm5644sup1.cif

Structure factors: contains datablock(s) I. DOI: 10.1107/S2056989022004844/wm5644Isup2.hkl

CCDC reference: 2170854

Additional supporting information:  crystallographic information; 3D view; checkCIF report

## Figures and Tables

**Table 1:** Experimental details

Crystal data	
Chemical formula	LaTe_1.90_
*M*_r_	381.35
Crystal system, space group	Tetragonal, *P*4_2_/*n*
Temperature (K)	100
*a*, *c* (Å)	10.1072 (3), 18.2874 (6)
*V* (Å^3^)	1868.16 (13)
*Z*	20
Radiation type	Mo *K*α
μ (mm^−1^)	25.70
Crystal size (mm)	0.11 × 0.08 × 0.04

Data collection	
Diffractometer	Bruker APEXII CCD
Absorption correction	Multi-scan (*TWINABS*; Bruker, 2012 ▸)
*T*_min_, *T*_max_	0.399, 0.749
No. of measured, independent and observed [*I* > 2σ(*I*)] reflections	7850, 7850, 5970
*R*_int_	0.055
(sin θ/λ)_max_ (Å^−1^)	1.000

Refinement	
*R*[*F* ^2^ > 2σ(*F* ^2^)], *wR*(*F* ^2^), *S*	0.027, 0.061, 1.12
No. of reflections	7850
No. of parameters	69
Δρ_max_, Δρ_min_ (e Å^−3^)	2.02, −3.08

Computer programs: *APEX2* and *SAINT* (Bruker, 2016 ▸), *SHELXT* (Sheldrick, 2015*a* ▸), *SHELXL* (Sheldrick, 2015*b* ▸), *DIAMOND* (Brandenburg, 2018 ▸) and *publCIF* (Westrip, 2010 ▸).

## supplementary crystallographic information

### Crystal data

**Table:**

LaTe_1.90_	*D*_x_ = 6.779 Mg m^−^^3^
*M**_r_* = 381.35	Mo *K*α radiation, λ = 0.71073 Å
Tetragonal, *P*4_2_/*n*	Cell parameters from 9906 reflections
*a* = 10.1072 (3) Å	θ = 3.6–46.4°
*c* = 18.2874 (6) Å	µ = 25.70 mm^−^^1^
*V* = 1868.16 (13) Å^3^	*T* = 100 K
*Z* = 20	Block, black
*F*(000) = 3116	0.11 × 0.08 × 0.04 mm

### Data collection

**Table:**

Bruker APEXII CCD diffractometer	7850 independent reflections
Radiation source: sealed X-ray tube	5970 reflections with *I* > 2σ(*I*)
Graphite monochromator	*R*_int_ = 0.055
φ and ω scans	θ_max_ = 45.3°, θ_min_ = 2.9°
Absorption correction: multi-scan (*TWINABS*; Bruker, 2012)	*h* = −13→14
*T*_min_ = 0.399, *T*_max_ = 0.749	*k* = 0→20
7850 measured reflections	*l* = 0→36

### Refinement

**Table:**

Refinement on *F*^2^	Primary atom site location: iterative
Least-squares matrix: full	Secondary atom site location: difference Fourier map
*R*[*F*^2^ > 2σ(*F*^2^)] = 0.027	*w* = 1/[σ^2^(*F*_o_^2^) + (0.0122*P*)^2^ + 6.7117*P*] where *P* = (*F*_o_^2^ + 2*F*_c_^2^)/3
*wR*(*F*^2^) = 0.061	(Δ/σ)_max_ = 0.002
*S* = 1.12	Δρ_max_ = 2.02 e Å^−^^3^
7850 reflections	Δρ_min_ = −3.08 e Å^−^^3^
69 parameters	Extinction correction: SHELXL-2016/6 (Sheldrick 2015b), Fc^*^=kFc[1+0.001xFc^2^λ^3^/sin(2θ)]^-1/4^
0 restraints	Extinction coefficient: 0.001129 (18)

### Special details

**Table:**

Geometry. All esds (except the esd in the dihedral angle between two l.s. planes) are estimated using the full covariance matrix. The cell esds are taken into account individually in the estimation of esds in distances, angles and torsion angles; correlations between esds in cell parameters are only used when they are defined by crystal symmetry. An approximate (isotropic) treatment of cell esds is used for estimating esds involving l.s. planes.
Refinement. Refined as a 2-component twin.

### Fractional atomic coordinates and isotropic or equivalent isotropic displacement parameters (Å^2^)

**Table:**

	*x*	*y*	*z*	*U*_iso_*/*U*_eq_	
La1	0.250000	−0.250000	0.38287 (2)	0.00695 (4)	
La2	0.14760 (2)	0.05231 (2)	0.10752 (2)	0.00710 (3)	
La3	−0.15137 (2)	−0.04353 (2)	0.38089 (2)	0.00696 (3)	
Te1	−0.250000	0.250000	0.43532 (2)	0.00722 (4)	
Te2	0.14951 (2)	0.05177 (2)	0.43621 (2)	0.00726 (3)	
Te3	−0.15255 (2)	−0.04952 (2)	0.07093 (2)	0.00722 (3)	
Te4	−0.250000	−0.250000	0.250000	0.00773 (5)	
Te5	−0.02702 (2)	0.16873 (2)	0.25027 (2)	0.00992 (3)	
Te6	0.06086 (2)	−0.12938 (2)	0.24930 (2)	0.00770 (3)	

### Atomic displacement parameters (Å^2^)

**Table:**

	*U*^11^	*U*^22^	*U*^33^	*U*^12^	*U*^13^	*U*^23^
La1	0.00683 (8)	0.00672 (8)	0.00729 (7)	0.00054 (6)	0.000	0.000
La2	0.00723 (6)	0.00691 (6)	0.00715 (5)	0.00000 (5)	0.00005 (4)	0.00083 (4)
La3	0.00657 (6)	0.00689 (6)	0.00744 (5)	0.00015 (5)	−0.00001 (4)	−0.00070 (4)
Te1	0.00701 (9)	0.00696 (9)	0.00769 (8)	0.00009 (7)	0.000	0.000
Te2	0.00684 (7)	0.00726 (7)	0.00767 (5)	−0.00005 (6)	0.00002 (5)	−0.00037 (4)
Te3	0.00712 (7)	0.00697 (7)	0.00756 (5)	0.00007 (6)	0.00001 (5)	0.00005 (5)
Te4	0.00815 (7)	0.00815 (7)	0.00690 (11)	0.000	0.000	0.000
Te5	0.01106 (7)	0.01167 (7)	0.00702 (6)	0.00045 (6)	0.00031 (6)	−0.00005 (5)
Te6	0.00881 (7)	0.00761 (6)	0.00667 (5)	0.00034 (5)	−0.00003 (5)	−0.00016 (5)

### Geometric parameters (Å, º)

**Table:**

La1—Te3^i^	3.2938 (2)	La2—Te6	3.2960 (2)
La1—Te3^ii^	3.2938 (2)	La2—Te1^ii^	3.3198 (2)
La1—Te1^iii^	3.3248 (3)	La2—Te5	3.3637 (3)
La1—Te6	3.3329 (3)	La3—Te1	3.2843 (2)
La1—Te6^iv^	3.3329 (2)	La3—Te4	3.3284 (2)
La1—Te2^iv^	3.3593 (2)	La3—Te3^vii^	3.3361 (3)
La1—Te2	3.3594 (2)	La3—Te6	3.3384 (2)
La1—Te5^ii^	3.4179 (3)	La3—Te2^iii^	3.3460 (2)
La1—Te5^i^	3.4179 (3)	La3—Te2	3.3465 (3)
La2—Te3^v^	3.2637 (2)	La3—Te3^ii^	3.3574 (3)
La2—Te2^vi^	3.2649 (3)	La3—Te6^vii^	3.4194 (3)
La2—Te3	3.2726 (3)	La3—Te5	3.4480 (3)
La2—Te2^i^	3.2920 (3)	Te5—Te6^vi^	2.9224 (3)
La2—Te5^i^	3.2944 (3)	Te5—Te6	3.1413 (3)
			
Te3^i^—La1—Te3^ii^	150.272 (10)	Te2^iii^—La3—Te3^ii^	73.330 (5)
Te3^i^—La1—Te1^iii^	75.136 (5)	Te2—La3—Te3^ii^	84.571 (6)
Te3^ii^—La1—Te1^iii^	75.136 (5)	Te1—La3—Te6^vii^	74.669 (6)
Te3^i^—La1—Te6	127.287 (6)	Te4—La3—Te6^vii^	59.908 (4)
Te3^ii^—La1—Te6	76.715 (5)	Te3^vii^—La3—Te6^vii^	72.455 (5)
Te1^iii^—La1—Te6	137.132 (4)	Te6—La3—Te6^vii^	89.696 (6)
Te3^i^—La1—Te6^iv^	76.715 (5)	Te2^iii^—La3—Te6^vii^	135.020 (7)
Te3^ii^—La1—Te6^iv^	127.288 (6)	Te2—La3—Te6^vii^	135.283 (7)
Te1^iii^—La1—Te6^iv^	137.132 (4)	Te3^ii^—La3—Te6^vii^	131.586 (6)
Te6—La1—Te6^iv^	85.735 (8)	Te1—La3—Te5	76.027 (6)
Te3^i^—La1—Te2^iv^	85.364 (5)	Te4—La3—Te5	90.060 (6)
Te3^ii^—La1—Te2^iv^	86.094 (5)	Te3^vii^—La3—Te5	135.955 (7)
Te1^iii^—La1—Te2^iv^	73.124 (5)	Te6—La3—Te5	55.118 (5)
Te6—La1—Te2^iv^	135.915 (6)	Te2^iii^—La3—Te5	134.911 (7)
Te6^iv^—La1—Te2^iv^	72.977 (5)	Te2—La3—Te5	72.489 (5)
Te3^i^—La1—Te2	86.094 (5)	Te3^ii^—La3—Te5	129.745 (6)
Te3^ii^—La1—Te2	85.363 (5)	Te6^vii^—La3—Te5	64.015 (6)
Te1^iii^—La1—Te2	73.123 (5)	La3—Te1—La3^viii^	144.714 (11)
Te6—La1—Te2	72.977 (5)	La3—Te1—La2^vii^	85.769 (5)
Te6^iv^—La1—Te2	135.915 (6)	La3^viii^—Te1—La2^vii^	86.028 (4)
Te2^iv^—La1—Te2	146.246 (10)	La3—Te1—La2^vi^	86.029 (4)
Te3^i^—La1—Te5^ii^	126.928 (6)	La3^viii^—Te1—La2^vi^	85.769 (4)
Te3^ii^—La1—Te5^ii^	76.389 (5)	La2^vii^—Te1—La2^vi^	152.703 (10)
Te1^iii^—La1—Te5^ii^	135.428 (4)	La3—Te1—La1^iii^	107.643 (5)
Te6—La1—Te5^ii^	65.244 (6)	La3^viii^—Te1—La1^iii^	107.643 (5)
Te6^iv^—La1—Te5^ii^	51.284 (5)	La2^vii^—Te1—La1^iii^	103.648 (5)
Te2^iv^—La1—Te5^ii^	71.386 (5)	La2^vi^—Te1—La1^iii^	103.648 (5)
Te2—La1—Te5^ii^	137.123 (7)	La2^i^—Te2—La2^vi^	86.685 (6)
Te3^i^—La1—Te5^i^	76.389 (5)	La2^i^—Te2—La3^iii^	104.300 (6)
Te3^ii^—La1—Te5^i^	126.928 (6)	La2^vi^—Te2—La3^iii^	105.511 (6)
Te1^iii^—La1—Te5^i^	135.428 (4)	La2^i^—Te2—La3	148.230 (7)
Te6—La1—Te5^i^	51.283 (5)	La2^vi^—Te2—La3	85.472 (6)
Te6^iv^—La1—Te5^i^	65.243 (6)	La3^iii^—Te2—La3	107.465 (6)
Te2^iv^—La1—Te5^i^	137.122 (7)	La2^i^—Te2—La1	85.364 (5)
Te2—La1—Te5^i^	71.387 (5)	La2^vi^—Te2—La1	149.063 (7)
Te5^ii^—La1—Te5^i^	89.144 (9)	La3^iii^—Te2—La1	105.424 (7)
Te3^v^—La2—Te2^vi^	76.566 (5)	La3—Te2—La1	85.737 (5)
Te3^v^—La2—Te3	78.875 (7)	La2^v^—Te3—La2	101.125 (7)
Te2^vi^—La2—Te3	88.008 (6)	La2^v^—Te3—La1^vi^	105.604 (7)
Te3^v^—La2—Te2^i^	75.263 (5)	La2—Te3—La1^vi^	86.313 (5)
Te2^vi^—La2—Te2^i^	86.496 (6)	La2^v^—Te3—La3^ii^	104.549 (6)
Te3—La2—Te2^i^	154.134 (7)	La2—Te3—La3^ii^	85.690 (6)
Te3^v^—La2—Te5^i^	141.133 (7)	La1^vi^—Te3—La3^ii^	149.757 (7)
Te2^vi^—La2—Te5^i^	125.814 (7)	La2^v^—Te3—La3^vii^	105.889 (6)
Te3—La2—Te5^i^	127.388 (7)	La2—Te3—La3^vii^	152.986 (8)
Te2^i^—La2—Te5^i^	75.182 (6)	La1^vi^—Te3—La3^vii^	86.610 (5)
Te3^v^—La2—Te6	141.795 (7)	La3^ii^—Te3—La3^vii^	87.422 (6)
Te2^vi^—La2—Te6	128.902 (6)	La3^vii^—Te4—La3^ii^	88.029 (7)
Te3—La2—Te6	74.873 (6)	La3^vii^—Te4—La3^ix^	121.144 (4)
Te2^i^—La2—Te6	127.073 (7)	La3^ii^—Te4—La3^ix^	121.144 (4)
Te5^i^—La2—Te6	52.645 (6)	La3^vii^—Te4—La3	121.144 (4)
Te3^v^—La2—Te1^ii^	75.603 (6)	La3^ii^—Te4—La3	121.145 (4)
Te2^vi^—La2—Te1^ii^	152.164 (7)	La3^ix^—Te4—La3	88.029 (7)
Te3—La2—Te1^ii^	87.208 (5)	Te6^vi^—Te5—Te6	175.690 (9)
Te2^i^—La2—Te1^ii^	85.964 (5)	Te6^vi^—Te5—La2^vi^	63.706 (6)
Te5^i^—La2—Te1^ii^	77.677 (6)	Te6—Te5—La2^vi^	118.432 (7)
Te6—La2—Te1^ii^	75.863 (6)	Te6^vi^—Te5—La2	120.999 (8)
Te3^v^—La2—Te5	141.908 (8)	Te6—Te5—La2	60.772 (5)
Te2^vi^—La2—Te5	73.230 (6)	La2^vi^—Te5—La2	133.008 (9)
Te3—La2—Te5	77.427 (6)	Te6^vi^—Te5—La1^vi^	62.856 (5)
Te2^i^—La2—Te5	124.638 (7)	Te6—Te5—La1^vi^	114.389 (7)
Te5^i^—La2—Te5	76.593 (7)	La2^vi^—Te5—La1^vi^	125.977 (7)
Te6—La2—Te5	56.276 (6)	La2—Te5—La1^vi^	82.945 (6)
Te1^ii^—La2—Te5	131.965 (7)	Te6^vi^—Te5—La3	116.808 (7)
Te1—La3—Te4	133.902 (7)	Te6—Te5—La3	60.669 (5)
Te1—La3—Te3^vii^	86.745 (5)	La2^vi^—Te5—La3	83.822 (6)
Te4—La3—Te3^vii^	73.231 (5)	La2—Te5—La3	120.748 (7)
Te1—La3—Te6	130.412 (7)	La1^vi^—Te5—La3	113.748 (7)
Te4—La3—Te6	60.731 (4)	Te5^i^—Te6—Te5	85.692 (9)
Te3^vii^—La3—Te6	133.378 (6)	Te5^i^—Te6—La2	63.650 (6)
Te1—La3—Te2^iii^	73.811 (6)	Te5—Te6—La2	62.951 (6)
Te4—La3—Te2^iii^	134.830 (7)	Te5^i^—Te6—La1	65.861 (5)
Te3^vii^—La3—Te2^iii^	74.503 (5)	Te5—Te6—La1	120.592 (7)
Te6—La3—Te2^iii^	135.279 (7)	La2—Te6—La1	128.888 (7)
Te1—La3—Te2	85.654 (5)	Te5^i^—Te6—La3	120.461 (7)
Te4—La3—Te2	132.068 (6)	Te5—Te6—La3	64.213 (5)
Te3^vii^—La3—Te2	147.001 (7)	La2—Te6—La3	126.382 (7)
Te6—La3—Te2	73.072 (6)	La1—Te6—La3	86.290 (6)
Te2^iii^—La3—Te2	72.535 (6)	Te5^i^—Te6—La3^ii^	121.325 (7)
Te1—La3—Te3^ii^	147.141 (7)	Te5—Te6—La3^ii^	122.568 (7)
Te4—La3—Te3^ii^	72.959 (5)	La2—Te6—La3^ii^	83.998 (6)
Te3^vii^—La3—Te3^ii^	84.626 (6)	La1—Te6—La3^ii^	116.758 (7)
Te6—La3—Te3^ii^	75.783 (6)	La3—Te6—La3^ii^	118.169 (7)

Symmetry codes: (i) −*y*+1/2, *x*, −*z*+1/2; (ii) *y*, −*x*−1/2, −*z*+1/2; (iii) −*x*, −*y*, −*z*+1; (iv) −*x*+1/2, −*y*−1/2, *z*; (v) −*x*, −*y*, −*z*; (vi) *y*, −*x*+1/2, −*z*+1/2; (vii) −*y*−1/2, *x*, −*z*+1/2; (viii) −*x*−1/2, −*y*+1/2, *z*; (ix) −*x*−1/2, −*y*−1/2, *z*.
